# Enhanced X-ray emission arising from laser-plasma confinement by a strong transverse magnetic field

**DOI:** 10.1038/s41598-021-87651-8

**Published:** 2021-04-14

**Authors:** Evgeny D. Filippov, Sergey S. Makarov, Konstantin F. Burdonov, Weipeng Yao, Guilhem Revet, Jerome Béard, Simon Bolaños, Sophia N. Chen, Amira Guediche, Jack Hare, Denis Romanovsky, Igor Yu. Skobelev, Mikhail Starodubtsev, Andrea Ciardi, Sergey A. Pikuz, Julien Fuchs

**Affiliations:** 1 Institute of Applied Physics, RAS, 46 Ulyanov Street, Nizhny Novgorod, Russia 603950; 2 Joint Institute for High Temperatures, RAS, 13 Bd.2 Izhorskaya st., Moscow, Russia 125412; 3 Department of Physics, Lomonosov Moscow State University, 1 Bd. 2 Leninskiye Gory, Moscow, Russia 119991; 4 LULI - CNRS, CEA, UPMC Univ Paris 06 : Sorbonne Université, Ecole Polytechnique, Institut Polytechnique de Paris, 91128 Palaiseau Cedex, France; 5 Sorbonne Université, Observatoire de Paris, PSL Research University, LERMA, CNRS UMR 8112, 75005 Paris, France; 6 LNCMI, UPR 3228, CNRS-UGA-UPS-INSA, Toulouse, 31400 France; 7 ELI-NP, “Horia Hulubei” National Institute for Physics and Nuclear Engineering, 30 Reactorului Street, 077125 Bucharest-Magurele, Romania; 8 Imperial College, London, SW7 2BW United Kingdom; 9 National Research Nuclear University “MEPhI”, 31 Kashirskoe shosse, Moscow, Russia 115409

**Keywords:** Laser-produced plasmas, Magnetically confined plasmas

## Abstract

We analyze, using experiments and 3D MHD numerical simulations, the dynamic and radiative properties of a plasma ablated by a laser (1 ns, 10$^{12}$–10$^{13}$ W/cm$^2$) from a solid target as it expands into a homogeneous, strong magnetic field (up to 30 T) that is transverse to its main expansion axis. We find that as early as 2 ns after the start of the expansion, the plasma becomes constrained by the magnetic field. As the magnetic field strength is increased, more plasma is confined close to the target and is heated by magnetic compression. We also observe that after $\sim 8$ ns, the plasma is being overall shaped in a slab, with the plasma being compressed perpendicularly to the magnetic field, and being extended along the magnetic field direction. This dense slab rapidly expands into vacuum; however, it contains only $\sim 2\%$ of the total plasma. As a result of the higher density and increased heating of the plasma confined against the laser-irradiated solid target, there is a net enhancement of the total X-ray emissivity induced by the magnetization.

## Introduction

The investigation of the dynamics of strongly magnetized high-energy-density (HED) plasmas is a topic that has been recently the subject of significant effort by many groups. Permitted by the advent of new experimental facilities capable of developing strong magnetic fields^1–3^, such investigations have led to major progress in diverse fields such as laboratory astrophysics^4–12^ or inertial confinement fusion (ICF). As for the latter, for example, via a reduction in electron thermal conductivity, it was shown that magnetization could increase the fuel ion temperature in ICF targets^13^. Magnetization of an ICF target could take place either by embedding conventional indirect-drive hohlraum targets with seed magnetic fields^13,14^, or in the magnetized liner inertial fusion approach^15–17^, where the external magnetic field is generated by a set of external coils^18^. Magnetization has also been shown to localize heat transport^19^, which improves laser beam propagation^20^ and should reduce the growth of Laser-Plasma-Instabilities (LPI) if the laser heating is uniform enough^21^. Moreover, the presence of a strong magnetic field could substantially reduce the growth of hydrodynamic instabilities^14,22,23^, thus enhance fuel compression; and a reduction in electron thermal conductivity, where it was shown that magnetization could increase the fuel ion temperature in ICF targets^13^. Overall, all of this would have the major benefit that, by easing the requirement for ignition, lower-cost laser drivers would be needed than in the case of traditional ICF schemes^24^. However, many questions pertaining to HED plasma dynamics in strong magnetic field still need to be addressed in order to properly design magnetized ICF experiments.

**Figure 1:**
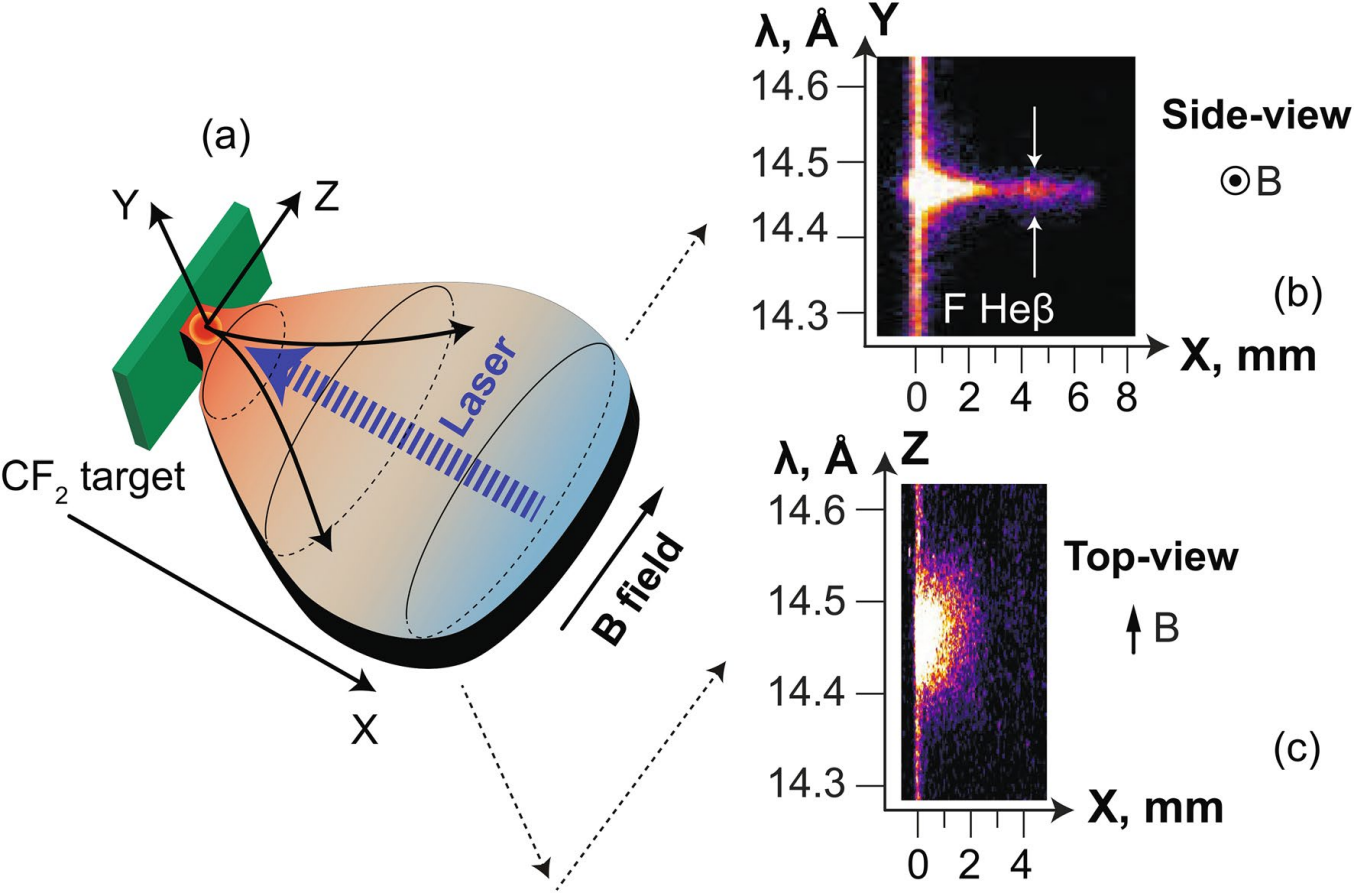
(**a**) Setup of the experiment and global plasma morphology resulting from the plasma-magnetic field interaction. A solid (Polytetrafluoroethylene (PTFE), CF$_2$) target, mimicking an ICF hohlraum wall, is immersed in a large-scale, steady, axial and strong (up to 30 T) magnetic field that is aligned with the target surface (along the z-axis). A long (0.6 ns FWHM) high-power (40 J, 4 × 10^13^ W/cm$^2$) laser pulse at $\lambda = 1$ µm irradiates the wall at normal incidence, inducing plasma heating and expansion. The laser was focused using a 2.2 m focal length lens (f/21) and a random phase plate^25^. (**b,c**) The images on the right represent the X-ray self-emission images of fluorine He$_{ \beta }$ spectral line obtained by viewing the 3D plasma side-on (i.e. in the XY plane) and from the top (i.e. in the XZ plane) where there was an 20 T applied magnetic field. These images are time-integrated and measured simultaneously on the same shot by two focusing spectrometers with spatial resolution (FSSR) deployed in the experiment (see “Methods” section). The images demonstrate that the global morphology of the plasma corresponds to that sketched in the cartoon (**a**), i.e. of a plasma that becomes extended in the XZ plane, while it is compressed in the XY plane. The white arrows in the side-view point to the increase in emissivity in the spatial region $\sim 4$ mm (see text).

Here we investigate, using experiments and 3D MHD numerical simulations, the hydrodynamic and radiative properties of a plasma expanding from a solid plane, and the impact on it of applying a strong (up to 30 T), homogeneous and steady magnetic field. This is done in a configuration where, as shown in Fig. 1, the magnetic (B) field is oriented parallel to the surface of the target, i.e. *transverse* to the main plasma propagation axis. This setup can represent a direct-drive ICF target outer surface or an indirect-drive ICF holhraum wall^26,27^ where the magnetic field is along the hohlraum axis. This configuration has been experimentally investigated for many years^28,29^ including in more recent studies^30–35^. These past studies focused mainly on the overall plasma dynamics over large distances, and revealed that the plasma morphology, as sketched in Fig. 1, under the influence of the transverse magnetic field, becomes that of a slab, i.e. compressed by the magnetic field in a thin layer in the XZ plane, with the plasma being able to extend along the magnetic field, i.e. along the Z-axis. Our detailed confirmation of the slab shape can be found in Ref.^34^. Note that this morphology is completely different from that induced by the magnetic field when it is *aligned* with the plasma main expansion axis, i.e. from that of a poloidal, or axial magnetic field. In that case, the plasma is collimated in all two-dimensions, thus forming a compact and dense “needle”-shaped column along the Z-axis^8,36,37^.

Here we will focus on the dynamics of the plasma expansion as illustrated in Fig. 1, with the aim to not characterize the long-range plasma propagation as was done before^30,31,33–35^, but the dynamics near the wall itself. Consistent with previous studies, we observe that in its initial launching at the target surface, the plasma has a ratio $\beta = p_{\text{plasma}}/p_{\text{magnetic field}} > 1$ (where $p_{\text{plasma}}$ is the overall plasma pressure, i.e. the sum of the ram and thermal pressures), and therefore is able to expand rapidly. However, as the plasma expands and cools down, $\beta$ becomes $<1$ at its leading edge, allowing the magnetic field to compress the plasma to form a cavity. This takes place already within 2 ns after the start of the expansion, i.e. over very short time scale compared to that typical of ICF compression experiment^24^. Regarding the self-generated Biermann battery magnetic field^38^, it will be constrained close to the ablation front via the Nernst effect^39,40^, and become negligible in the blow-off, compared to the strong externally applied magnetic field. Therefore, it is the external field that will guide the plasma dynamics in the slab. The compression is most efficient in the plane transverse to the field. As a result, the cavity emerges after $\sim$ 8 ns as a thin slab which propagates away from the target. However, this slab represents only a small fraction ($\sim$ 2$\%$) of the total plasma. Beyond this global behavior, our detailed analysis reveals that the magnetic field induces a strong separation between the two flows (a slow, dense one and a fast, low-density one) that are observed to stem from the wall. Moreover, the plasma is heated as it is compressed and focused onto the axis in one dimension. Overall, as a combined result of higher density and increased heating, we find that the plasma emissivity in the X-ray region is enhanced by at least 50$\%$ by the 30 T magnetization, compared to that of the unmagnetized plasma.

## Results

**Figure 2:**
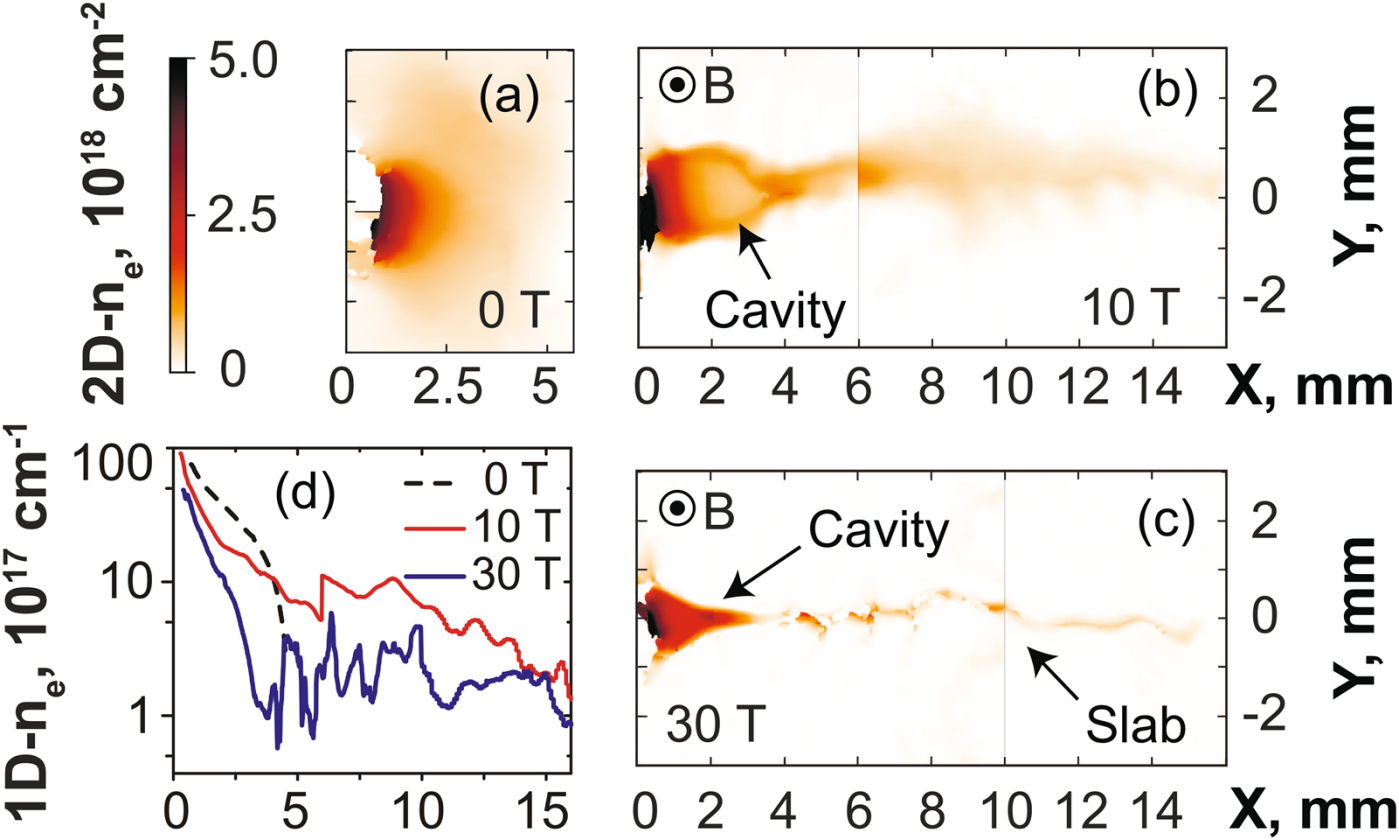
(**a–c**) 2D maps of the plasma electron density 40 ns after the laser irradiation on target (located on the left edge of the images at $x = 0$), for various strengths of the applied external magnetic field, as indicated. The color scale shown in (**a**) applies for all images. The images in (**b**,**c**) are reconstructed from two different shots with same parameters but obtained by moving the target within the diagnostic field of view, such that we can reconstruct a larger span of the plasma evolution. Patching this way two images obtained on two different shots is possible because the reproducibility of the results is very high, which is due to the external magnetic field generation having less than 1% variation from shot-to-shot^2^. (**d**) Corresponding radially integrated electron density as a function of the distance from the target, i.e. the 1D densities are obtained from 2D maps as shown in (**a**–**c**) and integrated over the axis Y. The lines correspond, respectively, to the case of a free expansion (black, dashed), 10 (thin red) and 30 T (thick blue). The maximum noise level is about $1 \times 10^{16}$ cm$^{-1}$, i.e. much lower than experimental data.

The experiment, which is shown in Fig. 1, was conducted on the ELFIE (LULI, Ecole Polytechnique, France) and TITAN (LLNL, USA) laser facilities. A high-power laser (see caption of Fig. 1 for details) irradiates at normal incidence a solid CF$_2$ planar target, from which a plasma is ablated and expands into vacuum (along the axis X). Note that using other laser incidence on target would have modified the laser absorption (but only weakly so, as long as the incidence angle is not large, i.e. typically over 50^41,42^), which would have thus changed the expanding plasma temperature and velocity, and thus the ratio between the expanding plasma pressure and the magnetic pressure. Thus, the length of the cavity that will be detailed below would have been affected, but otherwise, we do not expect that the overall magnetized plasma dynamics highlighted here would have been different. The laser-ablated plasma expands as well in the large-scale magnetic field, when it is applied. The magnetic field, which is directed along the axis Z, is generated by a modified Helmholtz coil^2^. It can be adjusted between 0–30 T on different shots, and can be considered, with respect to the scales of the expanding plasma, steady and homogeneous^36^. The plasma is diagnosed in several ways. First, the emission of the plasma in the X-ray domain is collected and analyzed using two time integrated instruments (see also the “Methods” section): a focusing spectrometer with spatial resolution (FSSR)^43,44^ and a variable line spaced grating spectrometer (VSG)^45^. The two are complementary: the VSG has a lower spectral resolution, but a higher bandwidth, than the FSSR. An image obtained by the FSSR of the plasma in the X–Y plane is shown in Fig. 1b as well as in Fig. 6a; the complementary image obtained in the X–Z plane is shown in Fig. 1c as well as in Fig. 6b. Second, an auxiliary optical laser probe is directed along the axis Z. Using an interferometry setup (we use the same setup as detailed in Ref.^6^), we measure with temporal resolution the low-density part of the plasma, as shown in Fig. 2a–c.

**Figure 3:**
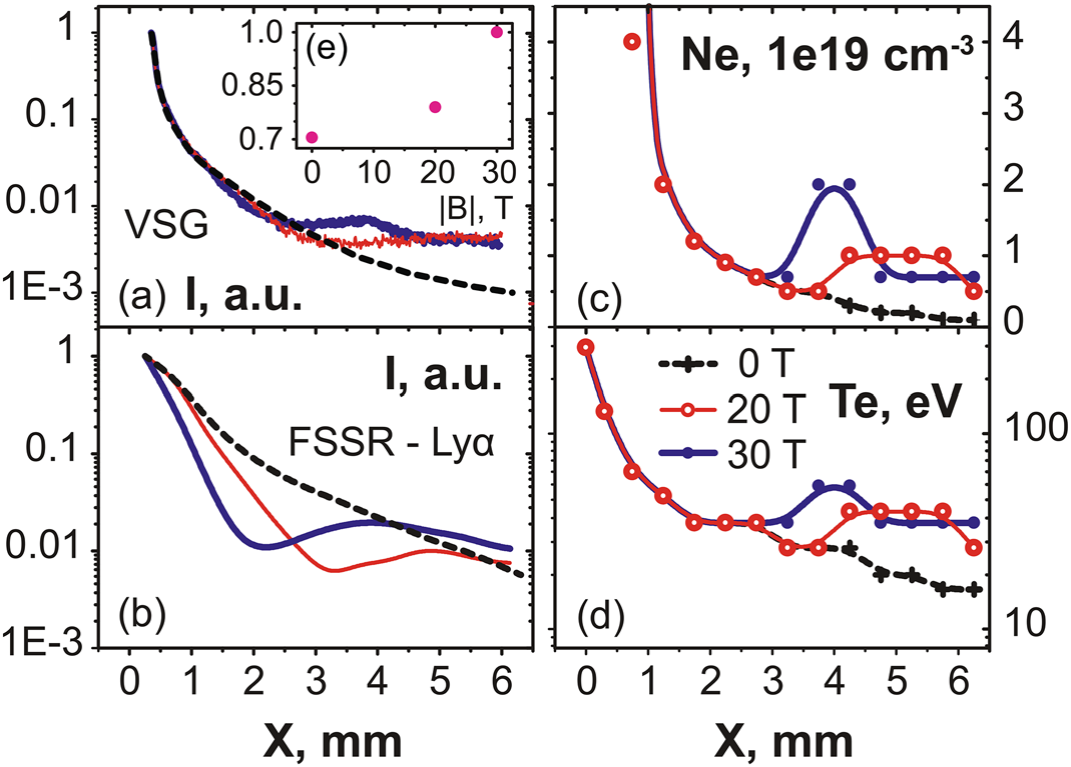
(**a**) Relative intensity profile measured by the VSG spectrometer in a wide spectral (integrated over 0.65–1 keV) and spatial range. (**b**) Relative intensity of the spectral line Ly$_{\alpha }$ measured by the FSSR spectrometer. (**c**) Profile of the volumetric electron density inferred in the plasma, and along the axis X, from the FSSR data (see text). (**d**) Same as (**c**) for the plasma electron temperature. For all panels, three cases, corresponding to different strengths of the applied magnetic field, are shown: 0 (black, dashed), 20 (red, thin) and 30 T (blue). Note that the values indicated in (**c**,**d**) are time-averaged due to time-integrated measurement performed by the FSSR spectrometer. (**e**) Evolution of the integrated plasma emission intensity (normalized to its value at $B = 30$ T), integrated in space (over the domain X = 1–6 mm) and time, and deduced from the intensity profiles shown in (**a**), as a function of the B-field strength.

**Figure 4:**
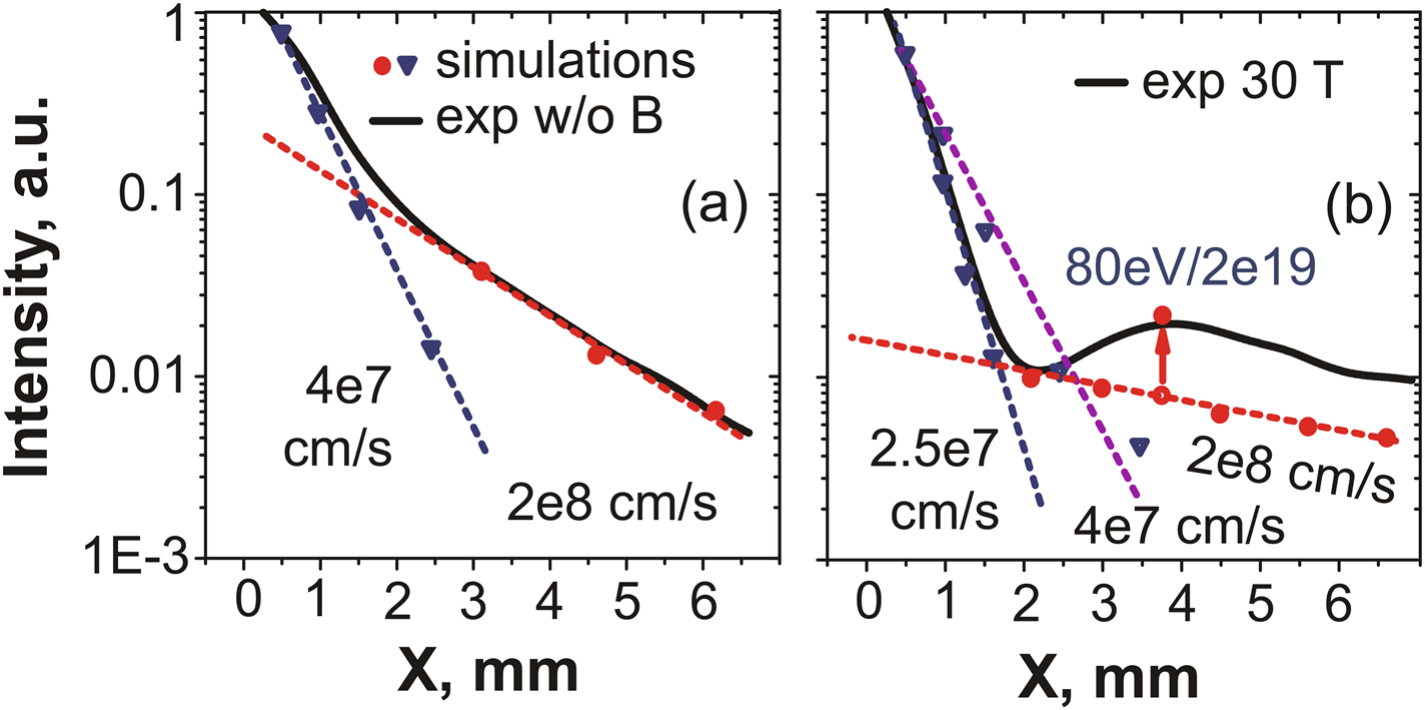
(**a**) Experimental (full) and simulated (dashed) spatial profiles of the X-ray resonance line Ly$_{\alpha }$ in the free expansion case. The theoretical intensities were normalized by the corresponding experimental points. (**b**) Same in the case where a transverse magnetic field $B=30$ T is applied. In the simulations, the excitation processes, as well as the recombination ones, were taken into account, as detailed in the “Methods” section. Note that the modelling, for the same velocity of the plasma component, but different densities can result in different slopes for the spectral intensity.

The impact of applying a transverse external magnetic field of increasing strength on the plasma propagation is shown in Fig. 2a–d, as retrieved by optical probing of the plasma, and Fig. 3, as retrieved from collecting the x-ray emission of the plasma. We briefly recall here the features of the unmagnetized plasma, i.e. a largely divergent expansion along the X-axis (see Fig. 2a) in which the plasma also cools down rapidly (see Fig. 3d). These features are further detailed in Ref.^36^ (see in particular Figs. 4 and 10 of that paper). In stark contrast, when applying the external magnetic field, we observe that the plasma is focused on axis by the magnetic tension into a conical shape, from which emerges a slab^33,34^ which is observed by the optical (Fig. 2b,c) and X-ray diagnostics (see Fig. 6a,b). Note that varying the laser intensity or energy on target, or the magnetic field strength, will affect the length and size of the cavity, but that the global morphology will stay the same, e.g. a larger deposited laser energy or a weaker magnetic field will result in a larger cavity^46^. From early snapshots of the interferometry diagnostics (not shown), we observe that the cavity already starts to form at $\sim 2$ ns, and that the slab emerges from the cavity at $\sim$ 8 ns. The slab is formed as the plasma is able to expand along the magnetic field lines (in the XZ plane), but is highly compressed and modulated^33,34^ in the XY plane (see Fig. 2b,c). This global morphology is consistent with previous observations^30,31,33,34^. Extending these previous studies, we can observe in Fig. 2b,c that the plasma compression in the XY plane increases with the magnetic field strength. At the same time, there is a noticeable decrease of the overall plasma mass flow in the region $x > 2$ mm that can be observed in Fig. 2d. Note that the optical probe is limited to measuring the plasma in the region $x > 1$ mm since beyond this, the optical probe is refracted by the dense plasma.

As the plasma flow is quenched by the magnetic field far away from the target, we would expect that this is linked to plasma being retained against the target. This is actually what is observed by the X-ray diagnostics, which complements the optical probing since they can resolve the plasma emission down to the target surface. Moreover, since the X-ray ion emission spectra are time-integrated, they offer the advantage of informing us on the global modification of the plasma flow under the influence of the external magnetic field, whereas the optical probing is limited to snapshots of the plasma evolution. Figure 3a compares the relative intensity of the plasma emission recorded in a broad X-ray energy range and for different strengths of the applied magnetic field, as recorded by the VSG spectrometer. We readily observe a much more constrained plasma emission profiles when increasing the magnetic field from 20 (red) to 30 T (blue), respectively. This can be observed consistently as well in the emission recorded by the FSSR (see Fig. 3b), which, due to its higher resolution allows us to further analyze separate plasma emission spectral lines (see Fig. 6a). In all spectral lines (as illustrated in Fig. 3b by the case of the Ly$_{\alpha }$ line), we clearly see that the relative intensity of the emission drops more steeply as the magnetic field strength is increased. We also observe that the X-ray spectral intensity increases again in the region 2–5 mm from the target surface, and even exceeds the intensity of the freely propagating plasma farther away. As shown in Fig. 3e, integrating in space and time the overall X-ray emission as recorded by the broadband VSG measurements shows that there is at least a 50$\%$ increase of the emission when the plasma is magnetized at 30 T compared to the unmagnetized plasma.

We will now discuss the plasma dynamics, as retrieved from the high-resolution X-ray data measured by the FSSR. Two techniques have been used. The first (detailed in the “Methods” section) is to fit the relative intensity of the lines present in the spectra. This was done for multiple line ratios^47^, and the inferred density was confirmed by the measurements of the interferometer. This yields the time-averaged, and local (volumetric) densities and temperatures in the plasmas. The results of this analysis, performed for various cases (unmagnetized and with 20 or 30 T applied), are shown in Fig. 3c,d. Note that in order to compare the volumetric densities measured by the FSSR to the line-integrated densities measured by optical interferometry, the plasma thickness along Z, i.e. along which the optical data are integrated, needs to be taken into account. To illustrate the comparison, let us consider the plasma at X = 6 mm in the 30 T magnetized case. The FSSR data shows an electron plasma density around $7 \times 10^{18}$ cm$^{-3}$ (see Fig. 3d). The interferometry data of Fig. 2c shows an integrated density around $1.3 \times 10^{18}$ cm$^{-2}$. Since indeed the length of the plasma along Z, as shown in Fig. 1c, is around 2 mm, this yields a volumetric density in the slab of around $6.5 \times 10^{18}$ cm$^{-2}$, i.e. consistent with the FSSR-retrieved density.

Obviously, very near the target surface, where the plasma ram pressure dominates the magnetic pressure, the plasma dynamics should be independent of the applied magnetic field. This is consistent with what we find, with the electron temperature being about 300 eV on the target surface in all cases (see Fig. 3d). Farther from the target, i.e. for $x > 3$–4 mm, we observe that the plasma temperature and volumetric density both increase when the transverse magnetic field is applied. Note that the density increase is consistent with what we observe in Fig. 2b,c with the optical probing, i.e. it corresponds to the tip of the cavity, where the plasma becomes strongly compressed by the magnetic field and forms the slab: as a result of the compression, the local density in the slab indeed increases. However, as highlighted by Fig. 2d, the global mass flow decreases with the increasing magnetic field. We also stress that the amount of plasma in the slab represents only $\sim 2\%$ (as deduced from the ratio of the density close to the target to that in the slab, see Fig. 2d) of all the volume of plasma.

To go beyond this first time-integrated analysis of the X-ray spectra and obtain a more refined picture of the plasma evolution close to the target surface, we simulate the spatial profile of spectral lines by solving the system of kinetic equations (detailed in the “Methods” section) governing the emission, and taking into account the processes of recombination and excitation in ions. This procedure was applied for two spectral lines of multicharged fluorine ions—Ly$_{\alpha }$ and He$_{\beta }$—both for the unmagnetized and 30 T magnetized cases. The results are shown in Fig. 4 for the Ly$_{\alpha }$ line, but similar results are obtained from the He$_{\beta }$ line (as shown in the “Methods” section), showing the robustness of the analysis. To be able to correctly fit the spatial profile of the line along X, we find that we have to model the plasma as composed of two plasma fractions, each having different velocities. Of course, the ionic distribution of the plasma in terms of velocity is actually a continuous function. However, as shown below in Fig. 4, we find that approximating such a continuous distribution function by a two-step, piece-wise function and therefore a two components plasma, in both in the unmagnetized and magnetized cases, leads to good fitting of the X-ray line spatial profile.

As shown in Fig. 4, what we find is that the first plasma component, that extends from the target surface to several millimeters, is rather slow, but contains most of the plasma. It is characterized by velocities up to $4 \times 10^7$ cm/s. The second one that extends beyond 4 mm from the target surface is fast, with velocities $\sim 10^8$ cm/s, but concerns only a low-density part of the plasma, i.e. it contains only 5–10$\%$ of the total number of particles. Note that these velocities are not thermal ones, but correspond to a ram motion of the plasma. They are consistent with those that can be derived from observing the progression of the plasma tip with the optical interferometer. Note also that when simulating independently the Ly$_{\alpha }$ and He$_{\beta }$ spectral lines, we obtained slightly different velocities for the fast and slow components (see Fig. 8), which can be explained by the inhomogeneity of charge distribution in the plasma, i.e. the hotter plasma having a higher percentage of bare nuclei.

We observe in Fig. 4 that the 30 T external magnetic field, when applied, has the notable effect of further separating the two inferred components. Compared to the unmagnetized case, most of the plasma finds itself confined by the magnetic field against the target, which translates into the averaged plasma velocity near the target becoming lower ($4 \times 10^7$ cm/s in the unmagnetized case vs $2.5 \times 10^7$ cm/s in the 30 T case). The fact that the slow, high-density and the fast, low-density components of the plasma are further separated in the presence of the external magnetic field can be easily understood. Indeed, the slow plasma component has an averaged velocity for ions with energies corresponding to the Larmor radius (about 0.3 mm) which does not exceed the transverse plasma size. This explains the increased plasma confinement by the magnetic field: the forward motion of these ions is slowed down by the magnetic field, which leads to an enhanced plasma emission near the target surface ($Z < 4$ mm). This is in contrast with the second plasma component for which the Larmor radius ($\sim 2$ mm) exceeds the transverse plasma size. These ions are associated to the plasma emission which is recorded far from the target surface ($Z \gg 4$ mm).

**Figure 5:**
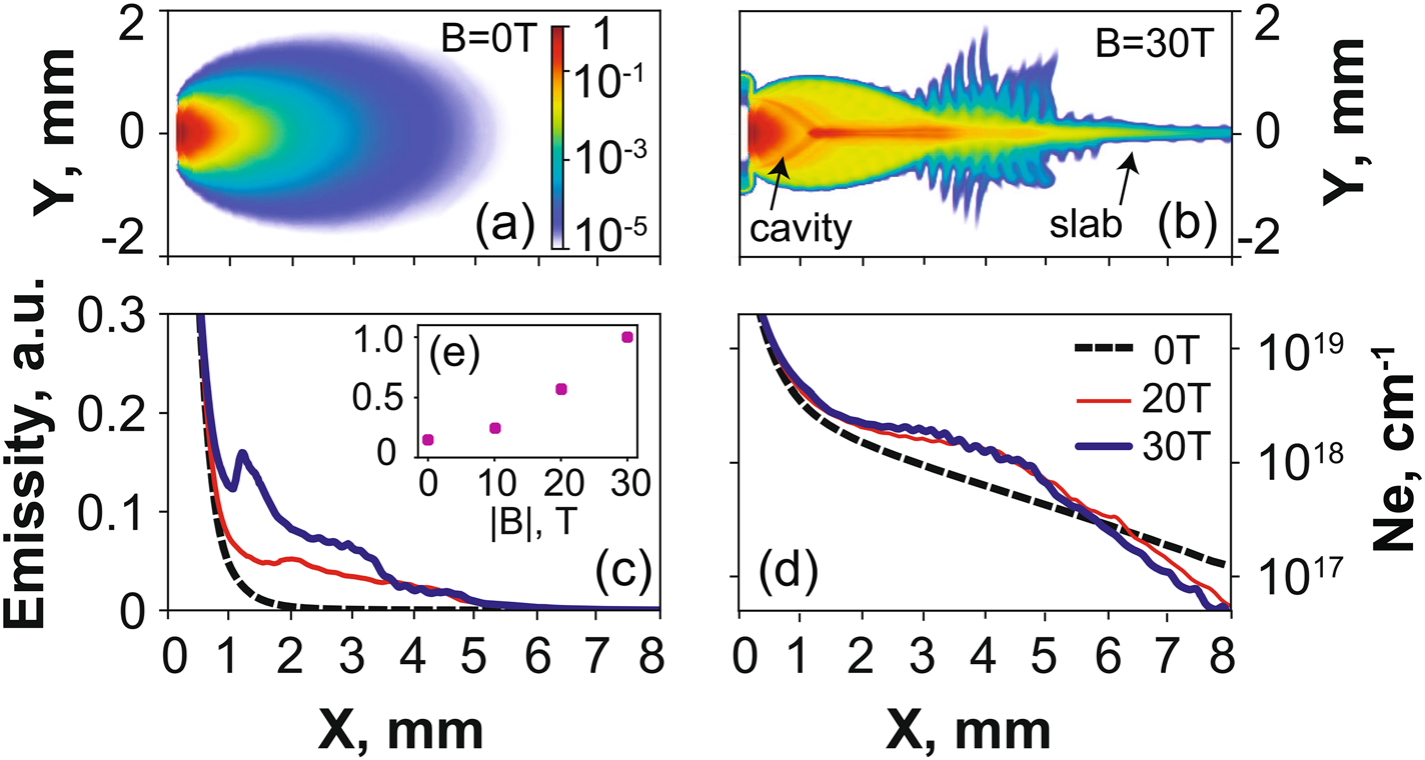
3D MHD simulation results. (**a**,**b**) Pseudocolor maps of the relative plasma emission intensity in the XY plane, in logarithm, and for (as indicated) $B = 0$ T and $B = 30$  T, respectively. Images are normalized to their own respective maximum, which is distinct for each case. (**c**) Profiles of relative emission intensity, integrated over time (up to 24 ns, which is the maximum time in the simulation) and over the Y and Z-directions, for $B = 0$ (black, dashed), 20 (red, thin) and 30 T (blue) (similarly as in Fig. 3a). (**d**) Profiles of electron density in logarithm scale, integrated over time and over the Y and Z-directions, for $B = 0$ (black, dashed), 20 (red, thin) and 30 T (blue). (**e**) Evolution of the integrated plasma emission intensity (normalized to its value at $B = 30$ T), integrated in space and time, as a function of the B-field strength.

The dynamics of the plasma confinement, retrieved from the experimental results detailed above, is further supported by 3D MHD simulations of the process performed using the GORGON code (see “Methods” section for details about the simulations). The results of these simulations, run in the case without magnetic field and with a 30 T magnetic field, are shown in Fig. 5. We can observe in Fig. 5a,b that the compression of a thin slab also occurs in the simulation, and that this compression leads to increased density (see also Fig. 5d) and temperature along the axis. Also, we observe in the simulation flute-like filaments structures, which grow rapidly (within a few ns) along the Y-direction (B-field is along Z-direction), as can be seen in Fig. 5b. These structures are proved to be generated by the magnetized Rayleigh–Taylor instability. More detailed analysis of this phenomenon, as made by our group, can be found in Ref.^34^. The density and temperature enhancement observed in the simulation when applying the magnetic field results, in turns, in higher emissivity of the plasma in the magnetized case (see also Fig. 5c). Overall, integrating the radiation in space and time , we find that the confinement induced by the B-field drives the emission of the plasma as the B-field increases (by a factor $\sim 6$, passing from 0 to 30 T), as can be seen in Fig. 5e. The larger increase recorded in the simulation compared to that measured by the VSG and shown in Fig. 3e could be due to the emissivity in GORGON being integrated over all photon energies.

## Discussion

In summary, our experiment demonstrates the ability to strongly impact the dynamics of a laser-driven plasma flow from a solid target by applying an external *transverse* magnetic field. In particular, we have shown that the magnetic field leads to a reduction of the overall plasma flow; and with a more precise analysis, we reveal the increased separation of the plasma into two components: a dense, slow one, increasingly confined against the target as the magnetic field increases, and a low-density, fast one, which is redirected on axis by the magnetic field. The compression applied onto the plasma by the magnetic field in the plane transverse to the latter (the XY plane here) induces an increase of the local plasma density and temperature, both of which drive an increase in the plasma X-ray emission. We have shown here results obtained with given laser parameters, but in the Appendix, one can also see that a similar conclusion can be reached in another (lower) intensity regime. Our observations of such increase of X-ray emission (of an hohlraum wall) could constitute additional interest for magnetized ICF.

However, the fact that the hotter fast plasma component, redirected by the magnetic field, could actually reach the ignition capsule earlier than in the unmagnetized case needs to be considered in the overall design of direct/indirect-drive magnetized ICF.

The platform used for the present study could also be relevant to astrophysical investigations. Plasma interaction with crossed magnetic fields is indeed a phenomenon that takes place in a wide variety of astrophysical environments like at the edge of disks surrounding forming young stars or when coronal mass ejections (CMEs)^48^ propagate away from a star^49^. Notably, the ability for plasma to propagate against a transverse magnetic field, as observed here, could be of interest to disk edge physics, namely by allowing removal of matter from the disk and cross the gap separating the disk from the star, thus participating in slowing down the disk as postulated by Shakura and Sunyaev^50^.

## Methods

### FSSR X-ray spectrometer

**Figure 6:**
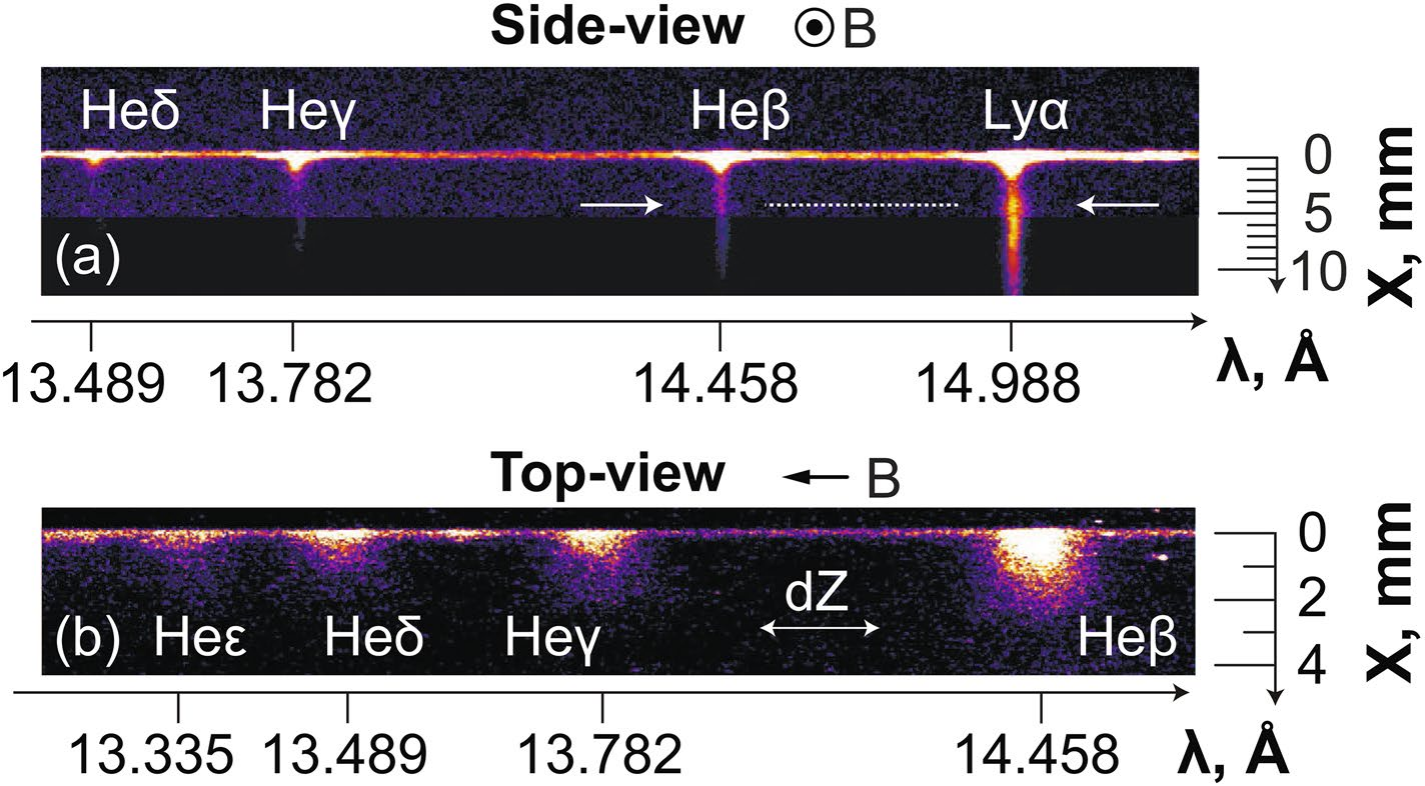
(**a**) Image of the X-ray emission analyzed by the FSSR spectrometer of the laser-induced plasma expansion inside the transverse magnetic field of 30 T strength. What is seen is the spectrally resolved image of the plasma in the XY plane. Arrows demonstrate the increase in emissivity in the spatial region $\sim 4$ mm. (**b**) FSSR X-ray raw image showing similar spectral range as in (**a**), but when probing the plasma along a different line of sight, here along the Y-axis of plasma expansion (see Fig. 1). Hence what is seen is the spectrally resolved image of the plasma in the XZ plane. The absence of the Ly$_{\alpha }$ line here is due to the different target-to-crystal distance that was changed to 214 mm in order to better observe the 2D dynamics (with higher spatial resolution along Z axis and magnification $m = 1$ in meridional plane) of the plasma expansion.

The FSSR is a X-ray Focusing Spectrometer with Spatial Resolution which uses a spherically bent mica crystal with $2d = 19.9149$ Å and curvature radius of $R=150$ mm for the mentioned experiment. This spectrometer allowed to observe plasma ion emission in the spectral range 13–16 Å with a spatial resolution about 0.1 mm along the X-axis and a spectral resolution better than $\lambda /d\lambda =1000$. Multicharged fluorine ions were analyzed through their hydrogen-like emission (transition 2p–1s) with dielectronic satellites and through the resonance series of helium-like ions (transitions 3p–1s, 4p–1s, 5p–1s etc.). Due to the presence of the magnetic field generating coil, the crystal was placed in a 2D scheme at a distance of 480 mm from the plasma source and the image plane was on the Rowland circle. Spectra were recorded using Fujifilm Image Plates of type TR, which were placed in a cassette holder protected from the optical radiation. The main spatial resolution of the spectrometer is along the X-axis. It is however also able to spatially resolve the plasma along the other axis (Y or Z in the experiment). Thus, the FSSR was able to observe the dynamics of the plasma expansion (see Fig. 6) which is fully consistent with what is observed by the optical interferometry probing (see Fig. 2a–d and Ref.^34^). The image in Fig. 6a was constructed from two different laser shots, the first being with the target located at the edge of the coil, in the field of view of the spectrometers, the second being with the target shifted within the coil, so that the plume farther from the target could be analyzed. This is possible since, due to the high reproducibility of the magnetic field generation, the expanding plasma dynamics is extremely reproducible^36^.

To retrieve the plasma parameters from the spectra recorded by the FSSR, the following procedure is applied. Near the target surface, i.e. for $x < 0.5$ mm, the relative intensities of the Ly$_{\alpha }$ line and of its dielectronic satellites were simulated using the radiative-collisional atomic code PrismSpect^51^ with a steady-state kinetic model. Farther from the target, the plasma is in a recombining mode^52^, so the quasi-stationary kinetic approach^47^ was rather used to retrieve the electron temperature and density profiles from the spectra. For this purpose, the relative intensities of He-like lines of fluorine (transitions 1s3p–1s2, 1s4p–1s2 etc.) were used^53^.

### VSG X-ray spectrometer

The variable line spaced grating spectrometer (VSG) is an X-ray spectrometer that allows to investigate a wider spectral range—200–2000 eV than the FSSR, but with a lesser spectral resolution^54^. It consists of a diffraction grating (1200 lpi in average), a spectrometer housing, and a removable nose cone that can house slits for spatially resolved measurements. In this experiment, the front of the housing was equipped with a vertical slit (i.e. aligned with the Y-axis), allowing to spatially resolve the plasma emission along the horizontal X-axis, which was the one of the plasma expansion. As for the FSSR, the image of the plasma was also recorded on a TR image plate, hence the measurement is time-integrated. In front of the image plate was a sheet of aluminized plastic to serve as a light-tight filter. For the deconvolution of the VSG data, the dependencies of the image plate sensitivity^55^ and of the reflectivity of the grating^56^ on the energy of photons were both taken into an account.

### Simulation of the experiment using the GORGON code

GORGON^57,58^ is a 3D, single-fluid, bi-temperature, resistive magneto-hydrodynamic (MHD) code. Originally developed to model Z-pinches and it has been widely used to model high-energy-density laboratory astrophysics experiments on lasers^8,34,36,59^. The average ionization charge is calculated using a local thermodynamic equilibrium Thomas–Fermi model, the plasma is assumed to be optically thin, and the radiation emission is computed assuming the dominant effect is due to recombination (free-bound) radiation^60^.

The simulation box is defined by a uniform Cartesian grid of dimension 8 mm $\times$ 8 mm $\times$ 12 mm and a number of cells equals to 400 $\times$ 400 $\times$ 600. The spatial resolution is homogeneous and its value is $dx = dy = dz =20$ $\upmu$m. The initial laser interaction with the solid target is performed using the DUED code^61^, which solves the single-fluid, 3-Temperature equations in two-dimensional axisymmetric, cylindrical geometry in Lagrangian form. The code uses the material properties of a two-temperatures equation of state (EOS) model including solid state effects, and a multi-group flux-limited radiation transport module with tabulated opacities. The laser-plasma interaction is performed in the geometrical optics approximation including inverse-Bremsstrahlung absorption.

The laser and target parameters in DUED are taken to be similar to the experimental ones, but there is here no magnetic field. At the end of the laser pulse (about 1 ns), the plasma profiles of density, momentum and temperature from the DUED simulations are used as initial conditions and remapped onto the 3D Cartesian grid of GORGON with a superimposed magnetic field. Note that the plasma expansion at this time is negligible with respect to the overall plasma expansion occurring during the whole experiment. The purpose of the hand-off between DUED and GORGON is to take advantage of the capability of the Lagrangian code to achieve very high resolution in modeling the laser-target interaction. The uniform magnetic field in GORGON is aligned with the target surface (in the Z-direction) and has magnitudes ranging from 10 to 30 T. We consider “outflow” boundary conditions. To remove the initial symmetry imposed by the 2D DUED simulations and to account for the effect of inhomogeneities in the laser intensity over the focal spot, we introduce uniformly distributed random perturbation on the plasma velocity components, with a maximum amplitude of $\pm 5\%$ the initial value. We note that this simulation method and in particular the initialization using the code DUED has been benchmarked in a variety of similar configurations^8,34,36,59^.

The simulations include anisotropic thermal conduction for the electrons and isotropic thermal conduction for the ions. The anisotropic thermal conduction is implemented in GORGON using a centered-symmetric algorithm^62,63^ with explicit super time stepping^64,65^. In general, for the plasma conditions explored here, the ions are always unmagnetized and anisotropic thermal conduction would be an unnecessary computational burden. The electrons instead have a Hall parameter which ranges from $\omega _{ce} \tau _e \lesssim 1$ close to the target, where collisionality is relatively high, to  10–100 in the slab. Thus heat fluxes are anisotropic mostly in the lower density regions of plume and the slab. However we note that in the plasma plume and slab the temperature gradients are relatively low and heat transport in these regions is generally dominated by the bulk plasma motions.

The simulations do not include the self-generation of magnetic field (Biermann battery) and thermally-driven magnetic field transport (Nernst advection), but, as shown below, these effects are unimportant under the plasma conditions found in our experiments. First of all we focus on the Biermann battery generated magnetic field. The ratio of the magnetic field induced by the plasma flow $\nabla \times (\overrightarrow {\user2{v}}\times \overrightarrow {\user2{B}})$ to the magnetic field generated by the Biermann battery, $(\nabla kT_{e}\times \nabla n_{e})/en_{e}$, can be approximated as:

$$\begin{aligned} \frac{\nabla \times (\overrightarrow {\user2{v}}\times \overrightarrow {\user2{B}})}{(\nabla kT_{e}\times \nabla n_{e})/en_{e}}\sim \frac{veB}{kT_{e}}L\sim \sqrt{\frac{Zm_{e}}{m_{i}}}\frac{L}{r_{L,e}}, \end{aligned}$$

where we have taken the flow velocity to be of the order of the ion sound speed ($c_S=\sqrt{(}Zk_BT_e/m_i)$), replaced the electron temperature $T_{e}=m_{e}v_{th,e}^{2}$, used the electron Larmor radius $r_{L,e}=v_{th,e}/\omega _{ce}$ and assumed that all gradients have a scale-length *L*. For an applied magnetic field $B=$ 10–30 T, and for the typical plasma conditions found in our experiments ($L=1$ mm and $T_{e}=300$ eV, $Z=8$), the ratio is $\sim$ 4–12, indicating that the advection term dominates over the Biermann battery term. We can also estimate the magnetic field that would be generated by the Biermann battery by considering the regime where it is being opposed by the convective transport (we are neglecting here the externally applied magnetic field). This then gives a saturated magnetic field (regime 3 of Haines^66^, see also simulations by Schoeffler et al.^67^):

$$\begin{aligned} B_{sat}\sim \frac{m_{i}}{Ze}\frac{c_{s}}{L}\sim 2.5\,\,\,\text {T}, \end{aligned}$$

which is well below the applied magnetic field, further strengthening the argument that the Biermann battery generated field is unimportant in our experiments. We note that the scaling for the saturated magnetic field, $B_{sat}\sim L^{-1}$, is valid for $L>d_i$, where $d_i$ is the ion inertial length, and it is applicable to our case, where $L/d_i \sim 100$. It is clear that the estimate above requires the density and temperature gradients to be maintained for a sufficiently long time in order to sustain the generation of the magnetic field up to the saturated value. While this is the case when the laser is on, these gradients are rapidly smoothed out when the laser is off, over a timescale of the order of $L/v_{th,e}<1$ ns^67^. Thus, in addition to being relatively small, the self-generation of magnetic field occurs over a relatively short time when compared to the plasma plume dynamics of tens of nanoseconds investigated in the experiments. Furthermore because of the convection of magnetic field by heat flow, the self-generated magnetic field is strongly localized within a few hundred microns from the target and relatively little magnetic field is present in the plasma plume^38,39,68,69^). Recent work also indicates that its strength in the plasma plume is also likely to be overestimated in simulations by 20–50% because of non-local heat transport^39,70^.

The simulations were run without the convection of magnetic field by heat flow (the Nernst effect). In general^71–73^, thermally-driven magnetic field transport occurs both perpendicular to the magnetic field at the Nernst velocity

$$\begin{aligned} \overrightarrow {\user2{v}}_N = - \frac{\beta _{\wedge }}{\omega _{ce}\tau _e}\frac{\tau _e}{m_e}\nabla k T_e, \end{aligned}$$

and perpendicular to both the magnetic field and the temperature gradient at the Nernst-cross-field velocity

$$\overrightarrow {\user2{v}}_{N,\wedge } = -\frac{\beta _{\parallel }-\beta _{\perp }}{\omega _{ce}\tau _e}\frac{\tau _e}{m_e} \hat{b}\times \nabla k T_e,$$

where $\beta$ are the components of the thermoelectric tensor^74,75^. Nernst advection dominates over the cross-field advection at low magnetization, while the opposite is true for $\omega _{ce}\tau _e > 1$. And their impact on magnetic field transport decreases with increasing magnetization, where for large Hall parameter^71^ $v_N \propto (\omega _{ce}\tau _e)^{-2}$ and $v_{N,\wedge } \propto (\omega _{ce}\tau _e)^{-1}$. These following estimates are obtained using the fits to the transport coefficients provided in Sadler et al.^74^. For our typical plasma conditions found in the plasma plume and slab ($B=10-30$ T, $Te \sim 10-300$  eV, $n_e \sim 10^{19}$ cm$^{-3}$, $T_e/|\nabla T_e|\sim 1$ mm) the Nernst and cross-field advection velocities (maximum $v_N\lesssim 10^6$ cm/s and $v_{N,\wedge }\lesssim 2\times 10^6$ cm/s) are always well below the corresponding ion sound speed or the typical plasma fluid velocities $10^7$–$10^8$ cm/s. This is also the case for the plasma closer to the target. For an upper estimate, taking a density $n_e \sim 10^{21}$ cm$^{-3}$ and assuming a steeper temperature gradient $\sim 100$ $\upmu$m, still gives a negligible Nernst velocity $\sim 10^6$ cm/s. It is clear however that the thermally driven transport of the applied magnetic field threading the laser focal spot, cannot be neglected early in the laser irradiation when the plasma is not set in motion yet. Assuming that over a short time $\delta t$ the plasma acquires a velocity $v \sim \delta t \nabla p / \rho$, the relative magnitude of hydrodynamics to Nernst advection velocity can then be estimated as^21^ $v_{\text {hydro}}/v_N = \delta t Z m_e / \tau _e m_i \gamma ^c_{\perp }$. These velocities will be of the same order over a times-scale $\delta t \sim \gamma ^c_{\perp } \tau _e \frac{m_i}{Zm_e}$, which for our plasma parameters corresponds to a time $\sim 10^{-10}$ s. The associated change in magnetic field due to the Nernst effect over this time-scale is $\Delta B \sim \delta t v_N B_{\text {applied}} / L < 1~\text {T}$. It is clear that all these estimates indicates that for our plasma conditions the transport of the magnetic field by the Nernst effect is negligible.

### Simulation of the relative intensities of the X-ray spectral lines registered by the FSSR spectrometer

**Figure 7:**
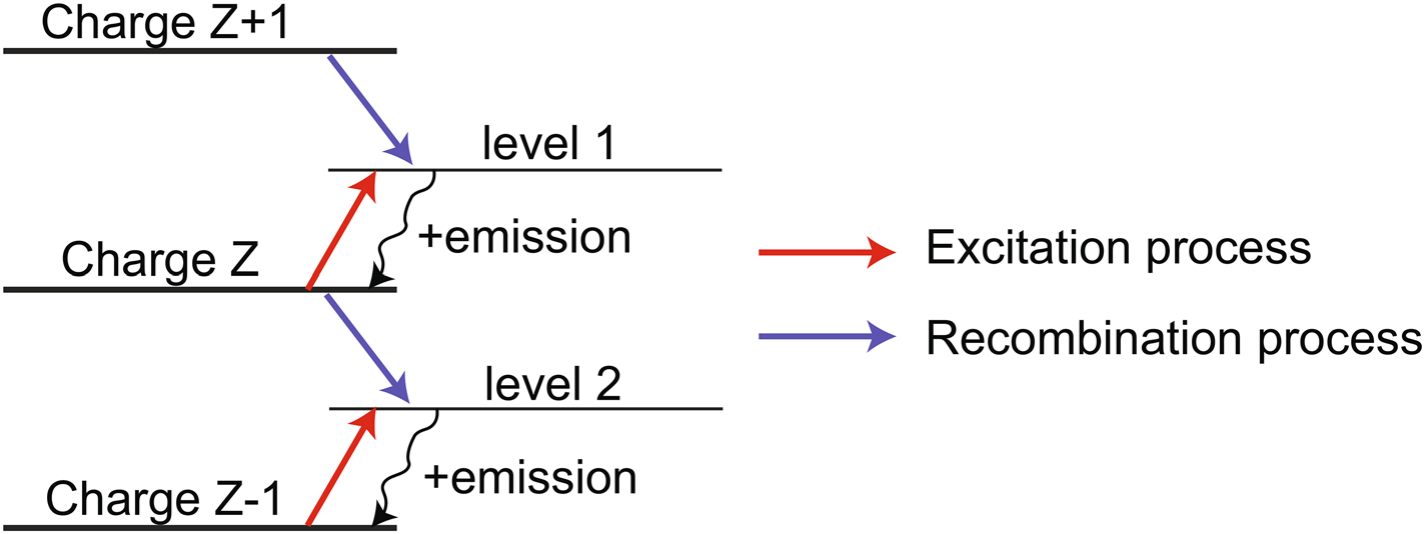
Scheme of the mechanisms producing the populations implied in the generation of the spectral resonance lines of multicharged ions. Here are depicted the levels with charges $Z-1$, *Z*, $Z+1$, each being in its ground states; levels 1 and 2 are excited levels. The red and blue lines correspond to the excitation or recombination population mechanism into the excited level. The further transition of an electron from each excited state (black arrows) leads to the ion emission of the corresponding spectral line.

Our second method to analyze the spatial profiles of the spectral lines recorded by the FSSR is detailed below. This approach is not only intrinsically refined compared to the first, time-averaged approach detailed above, but it allows also to better fit the spectral lines. Indeed, we can observe in Fig. 3c,d that the plasma density and temperature retrieved by the time-averaged approach varies monotonically close to the target surface ($x < 3$ mm) while obviously the intensity of the spectral lines (see Fig. 3b, and the arrows in Fig. 6a indicating the increase in intensity of the resonance lines) present strong variation in the same region, especially at 30 T. The time-dependent approach developed below was found to solve these issues and thus retrieve refined plasma parameters from the measurements. In general, the absolute intensity of the spectral line caused by the transition from the excited level *n* to the level *k* is known to be as follows:

1 $$\begin{aligned} I_{nk} = E_{nk} \cdot A_{nk} \cdot {N_n}^z, \end{aligned}$$

where E$_{\text{nk}}$ is the photon energy, A$_{\text{nk}}$ is a radiation probability, and ${{\hbox {N}_\text{n}}^\text{z}}$ is the population of corresponding ions with charge *Z* excited to the level *n*. The population of the excited states can be determined in a quasi-stationary approach by solving the system of kinetic equations and expressed through the population of ground states taking into account processes of recombination in ions with a charge $Z+1$ (see Fig. 7) and excitation in ions with charge *Z*^47^:

2 $$\begin{aligned} {N_n}^z = \beta _n \cdot N_e \cdot {N_1}^{z+1} + S_n \cdot {N_1}^z, \end{aligned}$$

where subscript 1 marks the ground state, N$_\text{e}$ is the electron density, $\beta _n$ and S$_\text{n}$ are population coefficients by the recombination and excitation, respectively. The population of the ground states is given by the following differential equations for the approximation of one-electron transitions:

3 $$\begin{aligned} \frac{{dN_1}^z}{dt} = \beta ^z \cdot {N_e}^2 \cdot {N_1}^{z+1} - (S^z \cdot N_e + \beta ^{z-1} \cdot {N_e}^2 ) \cdot {N_1}^z + S^{z-1} \cdot N_e \cdot N_1^{z-1}. \end{aligned}$$

Here $\beta ^z$ and $S^\text{z}$ are recombination and ionization rates for the processes $\hbox {A}_{\text{Z}+1} + 2\hbox {e} \rightarrow \hbox {A}_\text{Z} +\hbox {e}$, and $\hbox {A}_\text{Z} +\hbox {e} \rightarrow \hbox {A}_{\text{Z}+1} +2\hbox {e}$, respectively.

For the resonance line Ly$_{\alpha }$ (transition 2p–1s, *Z* corresponds here to H-like state) in the recombining plasma (i.e. the contribution of ionization is negligible), Eq. (3) can be easily modified to the following:

4 $$\begin{aligned} I_{21}(t) = E_{21} \cdot A_{21} \cdot \beta _2(N_e,T_e) \cdot N_e \cdot {N_1}^{z+1} (0) \cdot exp(-\int \limits _0^t \beta ^z(N_e,T_e) \cdot {N_e}^2dt), \end{aligned}$$

where ${\hbox {N}_1}^{\text{z}+1} (0)$ is the initial value of a bare nuclei concentration at time $t=0$. Here we consider the plasma to expand adiabatically^76,77^ with a fixed velocity $v=x/t$, where *x* is the distance from the target surface where the electron density drops as 1/e. It gives the opportunity to modify Eq. (4) with functions depending only on the distance, and to integrate them using the known dependences $N_e$(x), $T _e$(x), which are shown in Fig. 3c,d. Since the plasma parameters measured in the experiment can be considered as piecewise functions which are constant at certain spatial interval, then the spectral line intensity profile can be expressed for each interval *m* as an exponential function that has a dependence on the distance x from the target:

5 $$\begin{aligned} I_{21}(x) = E_{21} \cdot A_{21} \cdot \beta _2(m) \cdot N_e(m) \cdot {N_1}^{z+1}(m-1) \cdot exp(\beta ^z(m) \cdot {N_e}^2(m) \cdot (x_0(m)-x)/v), \end{aligned}$$

where $x_0(m)$ is the initial coordinate for each spatial interval. Hence, by now varying the velocity of the hydrodynamic flow, we can simulate the relative intensity of the resonance line Ly$_{\alpha }$ and thus retrieve the parameters of the plasma expanding freely in a vacuum as well as across the magnetic lines. The result is shown in Fig. 4. Note that, in our experiment, we consider the plasma to be optically thin due to parameters considered here, as discussed in details in Ref.^53^.

**Figure 8:**
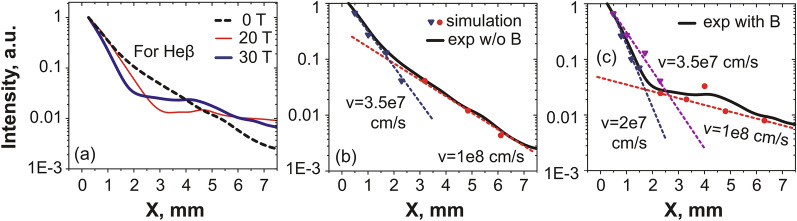
(**a**) Relative intensity of the spectral line He$_{\beta }$ measured by the FSSR spectrometer in the experiment both for a free expansion (black) and for the propagation in the transverse magnetic field with a strength of 20 (red) and 30 T (blue). (**b**) Experimental and simulated intensity profiles for the resonance line He$_{\beta }$ in the free expansion case. Theoretical intensities were normalized by the corresponding experimental point. The inferred plasma parameters are indicated. (**c**) Same as (**b**) but for the case when the transverse magnetic field $B = 30$ T is applied. Our analysis suggests an electron temperature of 54 eV at a density of $2\times 10^{19}$ $\hbox {cm}^{-3}$ in the shocked region.

**Figure 9:**
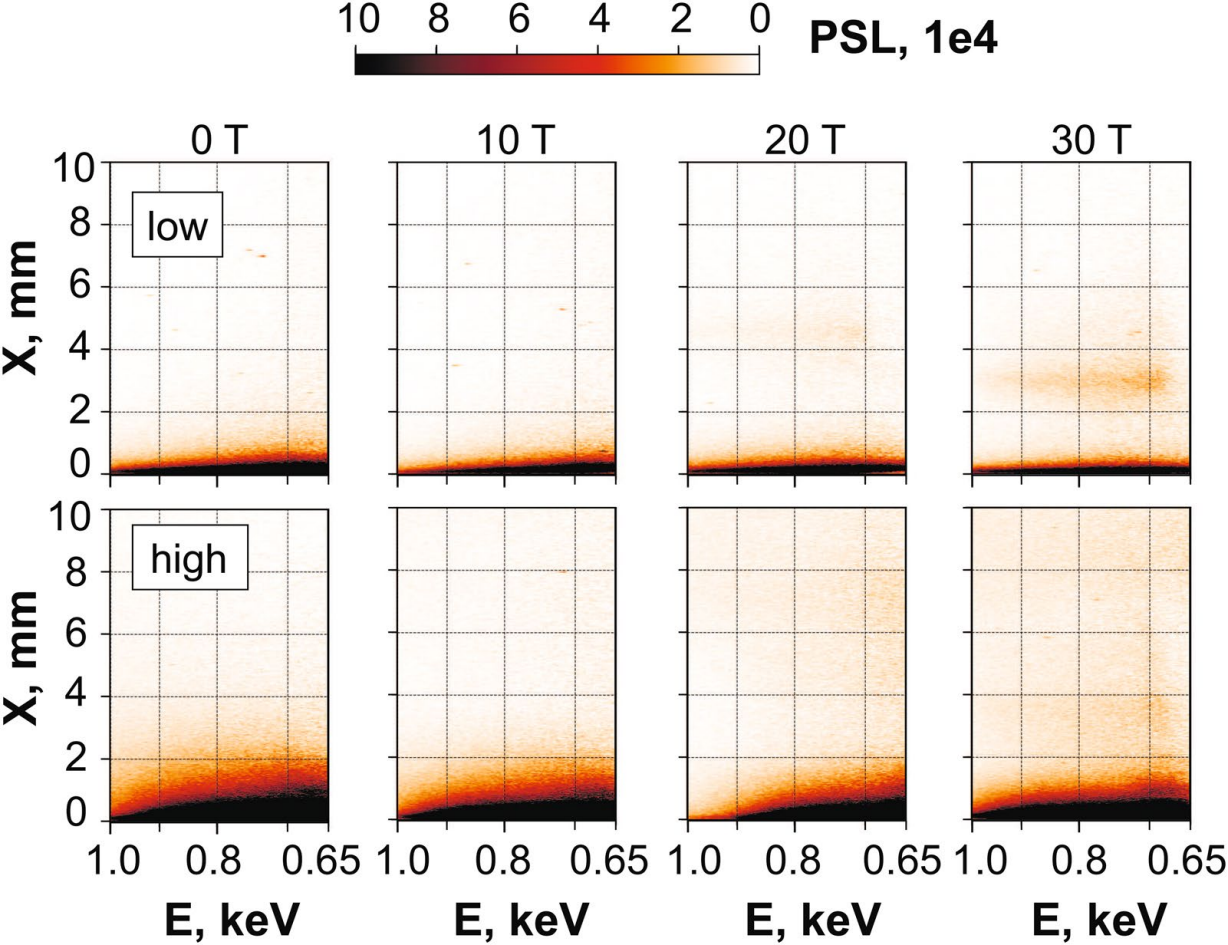
**(Top)** VSG X-ray images (in PSL) for a laser-induced plasma immersed into a magnetic field of different strengths 0–30 T and when the laser intensity is about $2 \times 10^{12}$ W/cm$^2$ on the target. **(bottom)** The same but for the laser intensity of $4 \times 10^{13}$ W/cm$^2$.

**Figure 10:**
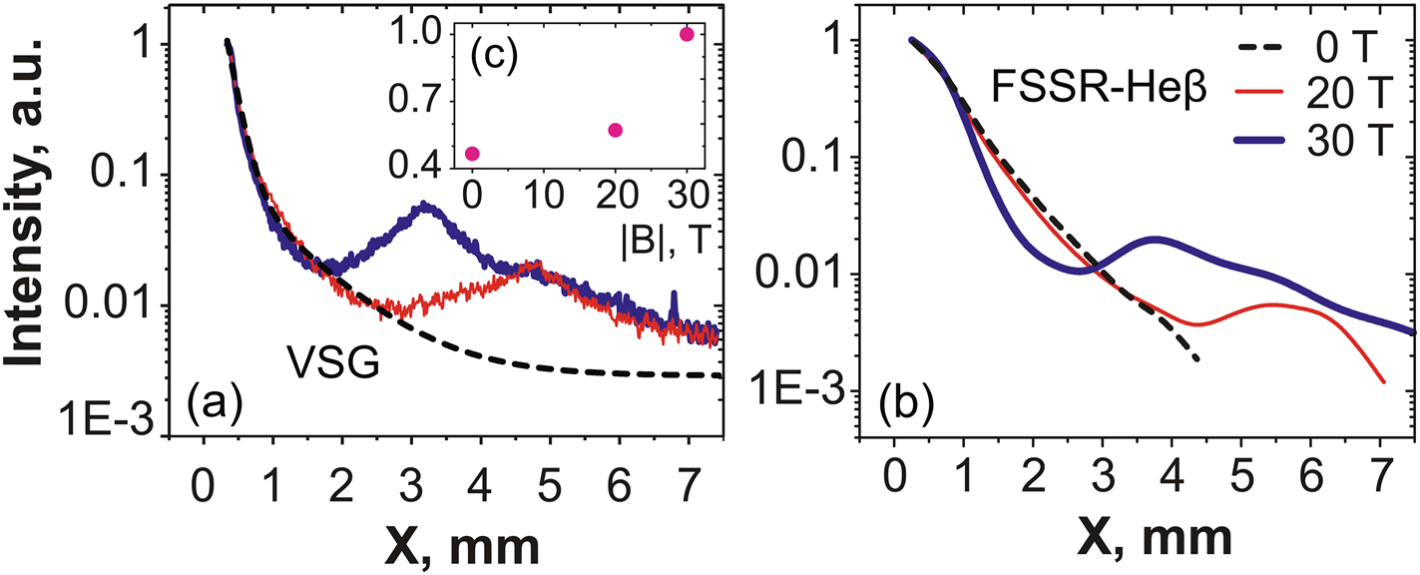
(**a**) Spatial profile of the emitted X-rays as recorded by the VSG and integrated over the 0.65–1 keV spectral range, for a $2 \times 10^{12}$ W/cm$^2$ laser intensity on target. The plasma was immersed into the magnetic field with strength of 30 T (blue), 20 T (red, thin) or expanded freely in vacuum (black dashed). (**b**) Same but for the He$_{\beta }$ spectral line measured by the FSSR spectrometer. (**c**) Evolution of the integrated plasma emission intensity (normalized to its value at $B = 30$ T), integrated in space and time, and deduced from the intensity profiles shown in (**a**), as a function of the B-field strength.

Similarly, the intensity of spectral lines other than Ly$_{\alpha }$ can be derived as well starting from Eq. (3). In order to verify the obtained data, the same technique was applied for the second intense spectral line in the investigated range—He$_{\beta }$, which measured spatial profiles of relative intensity, for the magnetized and unmagnetized cases, are shown in Fig. 8a. As a result of this procedure, the simulated relative intensity profiles, from which the plasma flow velocities of propagation are retrieved, are shown in Fig. 8b,c for, respectively, the unmagnetized and magnetized cases. We note that the plasma dynamics inferred from simulating the He$_{\beta }$ spectral line is similar to that obtained by the Ly$_{\alpha }$ line (see Fig. 4), which validates the robustness of the applied method. The slight differences in the retrieved plasma parameters, as inferred from the Ly$_{\alpha }$ and He$_{\beta }$ spectral lines, for the fast and slow components can be explained by the inhomogeneity of the charge distribution in the plasma, i.e. the hotter plasma having a higher percentage of bare nuclei.

We should note that, for the Ly$_{\alpha }$ line and in the spatial region close to $\sim 4$ mm, i.e. in the region where the plasma is being refocused and shocked^34^ by the magnetic field (see Fig. 3c,d), it is not possible to fully simulate the spatial variation in the line intensity (see the red arrow in Fig. 4b pointing to the discrepancy in amplitude between the simulated line intensity and the measured one). However, the simulated He$_{\beta }$ intensity profile fits the experimental line profile much more closely. It can be explained by the fact that Eq. (4) implies the plasma to be purely recombining. However, an additional plasma heating in the region x $\sim 4$ mm (which is valid for a higher ionic state), as due to the local strong focusing of the plasma induced by the magnetic field (see Fig. 5b), can lead to the situation when the excitation of the ground state of the H-like ion by electron collisions brings in additional contribution to the emissivity of the Ly$_{\alpha }$ line. In order to take into account the contribution of such excitation processes into the ion population, we used the fact that the intensity of He$_{\beta }$ line due to the recombination (transitions 1s–1s3p) and the intensity of Ly$_{\alpha }$ line due to the excitation (transitions 1s–2p) are based on the same concentration of H-like fluorine ions (see Fig. 7 with $Z = 8$, level 1 which corresponds to level 2p, level 2—1s3p). Then, considering Eq. (2), the intensity ratio of these lines can be evaluated as:

6 $$\begin{aligned} I(Ly_{\alpha })/I(He_{\beta }) = E(Ly_{\alpha })/E(He_{\beta }) \cdot A(Ly_{\alpha })/A(He_{\beta }) \cdot S_2(N_e,T_e)/(\beta _2(N_e,T_e) \cdot N_e). \end{aligned}$$

As a result, it gives the alternative measurement of the electron temperature about 80 eV at that location (close to $\sim 4$  mm) which corresponds to the measured intensity of the resonance line (shown in Fig. 4b). Note that the real spectral ratio $I(Ly_{\alpha })/I(He_{\beta })$ for the emission attributed to the shocked ionizing plasma at a particular time could be several times higher than the observed time-integrated value, and so the value of $T_e \sim 80$ eV should be considered as a lower estimation.

## Appendix: X-ray data for lower laser intensity (about 10$^{12}$ W/cm$^2$)

To complement the results shown in the paper ($4 \times 10^{13}$ W/cm$^2$), we here report on complementary measurements conducted at lower laser intensity, and which concur with the results obtained at higher intensity. To do this, we increased the size of the focal spot to reduce the intensity to about $2 \times 10^{12}$ W/cm$^2$ (20 times less than the nominal intensity). The comparison between the cases with “high” and “low” laser intensities is presented in Fig. 9 for a plasma immersed into the magnetic field with 0–30 T strength and as measured by the VSG spectrometer after taking into account all instrumental functions. Obviously, the total emissivity of the plasma is decreased near the target in the low intensity case, however, the spatial profile of the emissivity (see Fig. 10a) is similar to that recorded in the high intensity case (see Fig. 3a). Notably, as shown in Fig. 10c, we also observe in this reduced laser intensity case a net increase (by $\sim 50\%$ as well) of the overall X-ray emission in the 30 T magnetized case compared to that of the unmagnetized case. The FSSR data is also demonstrating the same dynamics of the plasma expansion in the transverse magnetic field as in the higher intensity case (compare Fig. 10b to Fig. 3b), although the intensity of the spectral lines is here quite low far from the target, which makes it difficult to measure the plasma parameters there.
